# Role of PAF Receptor in Proinflammatory Cytokine Expression in the Dorsal Root Ganglion and Tactile Allodynia in a Rodent Model of Neuropathic Pain

**DOI:** 10.1371/journal.pone.0010467

**Published:** 2010-05-03

**Authors:** Shigeo Hasegawa, Yuta Kohro, Miho Shiratori, Satoshi Ishii, Takao Shimizu, Makoto Tsuda, Kazuhide Inoue

**Affiliations:** 1 Department of Molecular and System Pharmacology, Graduate School of Pharmaceutical Sciences, Kyushu University, Fukuoka, Japan; 2 Department of Biochemistry and Molecular Biology, Faculty of Medicine, The University of Tokyo, Tokyo, Japan; Southern Illinois University School of Medicine, United States of America

## Abstract

**Background:**

Neuropathic pain is a highly debilitating chronic pain following damage to peripheral sensory neurons and is often resistant to all treatments currently available, including opioids. We have previously shown that peripheral nerve injury induces activation of cytosolic phospholipase A_2_ (cPLA_2_) in injured dorsal root ganglion (DRG) neurons that contribute to tactile allodynia, a hallmark of neuropathic pain. However, lipid mediators downstream of cPLA_2_ activation to produce tactile allodynia remain to be determined.

**Principal Findings:**

Here we provide evidence that platelet-activating factor (PAF) is a potential candidate. Pharmacological blockade of PAF receptors (PAFRs) reduced the development and expression of tactile allodynia following nerve injury. The expression of PAFR mRNA was increased in the DRG ipsilateral to nerve injury, which was seen mainly in macrophages. Furthermore, mice lacking PAFRs showed a reduction of nerve injury-induced tactile allodynia and, interestingly, a marked suppression of upregulation of tumor necrosis factor α (TNFα) and interleukin-1β (IL-1β) expression in the injured DRG, crucial proinflammatory cytokines involved in pain hypersensitivity. Conversely, a single injection of PAF near the DRG of naïve rats caused a decrease in the paw withdrawal threshold to mechanical stimulation in a dose-dependent manner and an increase in the expression of mRNAs for TNFα and IL-1β, both of which were inhibited by pretreatment with a PAFR antagonist.

**Conclusions:**

Our results indicate that the PAF/PAFR system has an important role in production of TNFα and IL-1β in the DRG and tactile allodynia following peripheral nerve injury and suggest that blocking PAFRs may be a viable therapeutic strategy for treating neuropathic pain.

## Introduction

Neuropathic pain that occurs after nerve injury results from an aberrant functioning of a pathologically altered nervous system [1], [2]. A hallmark of neuropathic pain syndrome is tactile allodynia, an abnormal hypersensitivity to innocuous stimuli, which is often resistant to all treatments currently available, including potent analgesic opioid drugs. The underlying mechanisms by which nerve injury develops tactile allodynia have remained largely unknown. The dorsal root ganglion (DRG) contains cell bodies of primary afferent neurons that transmit sensory information from the periphery to the central nervous system. The activation of signal transduction cascades and the transcriptional changes in the DRG and the resultant alterations in the transmission properties of sensory neurons following peripheral nerve injury might be involved in modulation of pain signaling in acute and chronic pain conditions [2], [3].

We have previously shown that peripheral nerve injury induces activation of cytosolic phospholipase A_2_ (cPLA_2_), a Ca^2+^-dependent subclass of the PLA_2_ family [4] that is required for tactile allodynia [5], in DRG neurons. However, the way in which activated cPLA_2_ participates in tactile allodynia remains unknown. cPLA_2_ is a crucial enzyme that catalyzes the hydrolysis of phospholipids to release arachidonic acid and lysophospholipid, and subsequently generates lipid mediators. Arachidonic acid is metabolized to prostaglandins by the cyclooxygenase (COX) pathway and to leukotrienes by the lipoxygenase (LOX) pathway. Lysophospholipid can be converted to platelet-activating factor (PAF) by lyso-PAF acetyltransferase and to lysophosphatidic acid (LPA) by lysophospholipase D. It raises the possibility that these lipid mediators mediated by cPLA_2_ activation may be secreted from DRG neurons and, in turn, may modulate the excitation of DRG neurons directly or indirectly. Indeed, prostaglandins have been shown to cause sensitization of peripheral sensory neurons (peripheral sensitization) [6] and to produce allodynic behavior [7], [8]. LOX products activate capsaicin receptors in primary sensory neurons, resulting in the induction of peripheral sensitization [9], [10]. Furthermore, PAF injected into the hindpaw of naïve animals produces nociceptive responses and mechanical hypersensitivity [11], and recent works have also shown that intrathecal administration of LPA [12] and PAF [13], [14] in naïve animals induces tactile allodynia. However, the role of these lipid mediators in the pathogenesis of neuropathic pain is not fully understood.

In the present study, to determine the neuropathic pain-related lipid mediators downstream of cPLA_2_ activation in the DRG, we investigate the involvement of enzymes and lipid mediator receptors in nerve injury-induced tactile allodynia using pharmacological, molecular, and genetic approaches. We further investigated the role of the lipid mediator receptors in the expression of tumor necrosis factor α (TNFα) and interleukin-1β (IL-1β) in the DRG, proinflammatory cytokines that are strongly implicated in nerve injury-induced tactile allodynia [15], [16], [17], [18].

## Results

### Effects of COX and LOX inhibitors on the development of tactile allodynia after nerve injury

**Figure 1:**
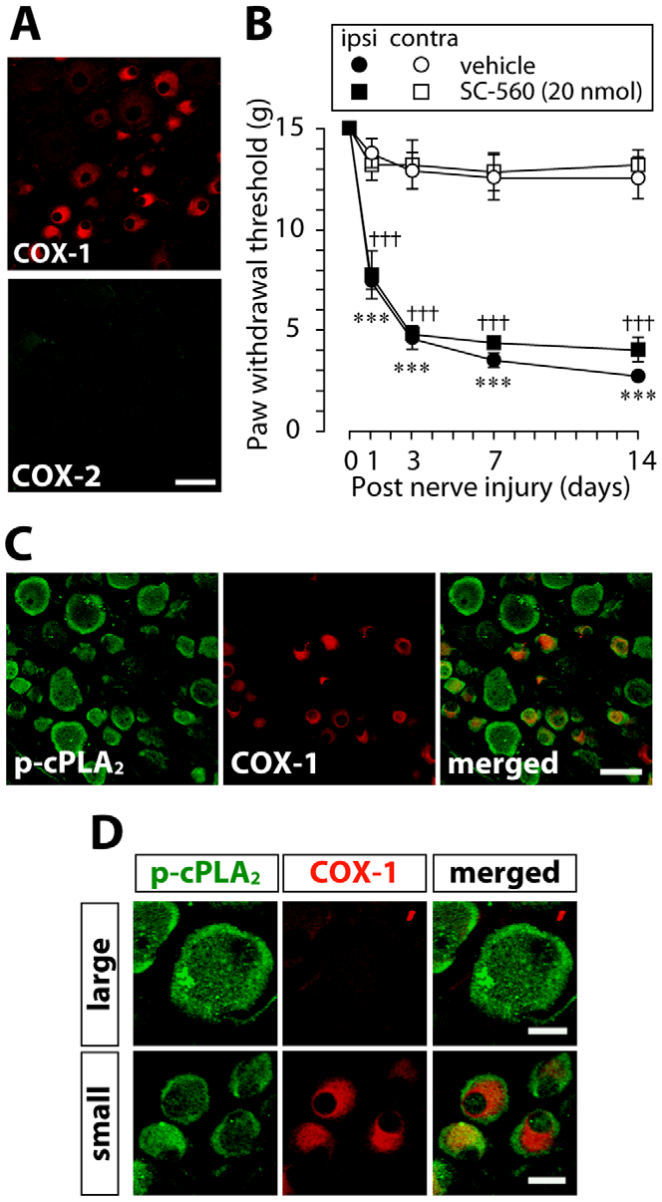
A COX inhibitor does not prevent the development of tactile allodynia after nerve injury. A. Immunofluorescence labeling of COX-1 and COX-2 proteins in the L5 DRG 14 days after nerve injury demonstrated that immunoreactivity of COX-1, but not COX-2, was present mainly in small-sized neurons. Scale bar, 50 µm. B. The COX-1 inhibitor SC-560 (20 nmol/10 µl) administered through a catheter whose tip was positioned near the L5 DRG once daily for 14 days did not suppress the development of nerve injury-induced a decrease in the paw withdrawal threshold of tactile stimulation using von Frey filaments. ***p<0.001 compared with the threshold of the vehicle-treated group on day 0. †††*p*<0.001 compared with the threshold of the inhibitor-treated group on day 0. Data are presented as mean ± SEM of the paw withdrawal threshold of five animals. C. Double immunofluorescence labeling of p-cPLA_2_ with COX-1 in L5 DRG neurons 14 days after nerve injury showed that most of neurons with the translocated p-cPLA_2_ were not double-labeled with COX-1-IR. Scale bar, 50 µm. D. Highly magnified pictures of large and small diameter DRG neurons demonstrated that in large neurons, p-cPLA_2_ was not co-expressed with COX-1-IR. In small neurons, the subcellular localizations of p-cPLA_2_ and COX-1-IR were different: COX-1-immunoreactivity was observed in the perinuclear area where p-cPLA_2_- immunoreactivity was not accumulated. Scale bars, 20 µm.

**Figure 2:**
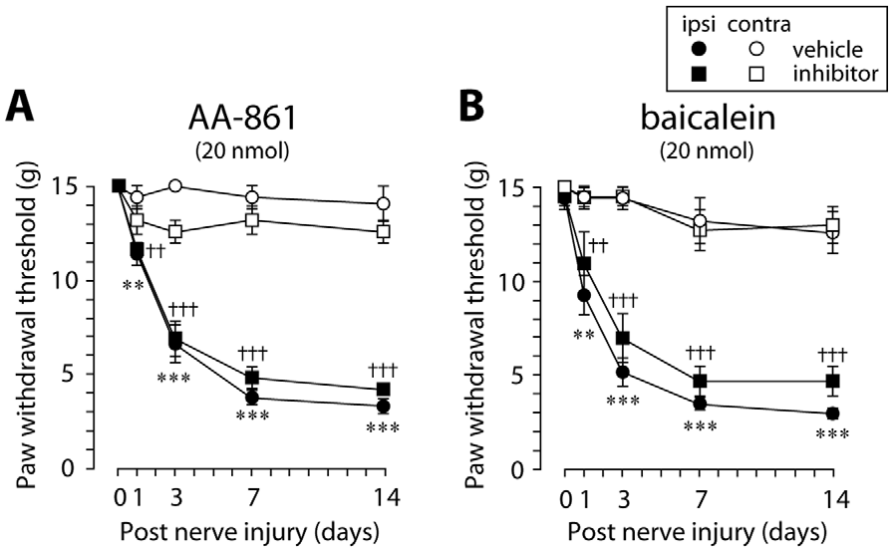
LOX inhibitors do not prevent the development of tactile allodynia after nerve injury. A, B. The 5-LOX inhibitor AA-861 (20 nmol/10 µl) (A) and the 12- and 15-LOX inhibitor baicalein (20 nmol/10 µl) (B) administered through a catheter whose tip was positioned near the L5 DRG once daily for 14 days did not suppressed the development of nerve injury-induced tactile allodynia. **p<0.01, ***p<0.001 compared with the threshold of the vehicle-treated group on day 0. ††*p*<0.01, †††*p*<0.001 compared with the threshold of the inhibitor-treated group on day 0. All data are presented as mean ± SEM of the paw withdrawal threshold of five animals.

To determine an involvement of a COX-dependent pathway in tactile allodynia, we first performed double-immunolabeling for phosphorylated-cPLA_2_ (p-cPLA_2_) and COX. In the both side of the L5 DRG after nerve injury, COX-1-immunoreactivity (COX-1-IR) was present mainly in small-sized neurons, whereas no COX-2-IR was observed (Fig. 1A), consistent with previous studies [19], [20]. Thus, we examined the effect of the selective COX-1 inhibitor SC-560 [21] on the development of nerve injury-induced tactile allodynia. Vehicle-treated rats with an L5 nerve injury displayed a marked decrease in paw withdrawal threshold at the ipsilateral side after nerve injury (*p*<0.001) (Fig. 1B). Similarly, nerve-injured rats that had been treated with SC-560 showed a decreased paw withdrawal threshold (*p*<0.001) and there were no significant differences in the threshold of ipsilateral side between the vehicle- and SC-560-treated group (Fig. 1B). We also found that most of neurons with the translocated p-cPLA_2_ were not double-labeled with COX-1-IR (169/251, 67.3% of p-cPLA_2_-translocated neurons) (Fig. 1C). In a small group of DRG neurons, especially small-sized neurons, both types of immunoreactivity were observed, but their subcellular localizations were different: COX-1-IR was observed in the perinuclear area where p-cPLA_2_-IR was not accumulated (Fig. 1D). Next, to investigate the involvement of LOX in nerve injury-induced tactile allodynia, we examined the effects of the 5-LOX inhibitor AA-861 [22] and the 12- and 15-LOX inhibitor baicalein [23], [24] and found that AA-861- and baicalein-treated rats displayed a decrease in paw withdrawal threshold with a similar time course to that of vehicle-treated rats (Fig. 2A, B). There were no significant differences in the threshold between the vehicle- and the LOX inhibitor-treated groups. The doses of the COX and LOX inhibitors used in this study are approximately equivalent to (or even higher than) doses that are known to inhibit behavioral responses mediated by COX [25], [26] and LOX [27], [28], respectively. Thus, these results suggest that COX and LOX in the DRG may not be involved in nerve injury-induced tactile allodynia.

### Effects of PAFR and LPAR antagonists on nerve injury-induced tactile allodynia

**Figure 3:**
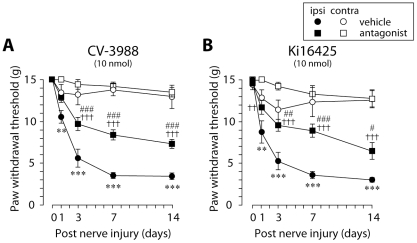
PAFR and LPAR antagonists suppress the development of tactile allodynia by injury to the L5 spinal nerve. A, B. Administration of PAFR antagonist CV-3988 (10 nmol/10 µl) (A) and the LPAR antagonist Ki16425 (10 nmol/10 µl) (B) through a catheter whose tip was positioned near the L5 DRG once daily for 14 days after nerve injury suppressed the development of tactile allodynia. **p<0.01, ***p<0.001 compared with the threshold of the vehicle-treated group on day 0. ††*p*<0.01, †††*p*<0.001 compared with the threshold of the antagonist-treated group on day 0. #*p*<0.05, ##*p*<0.01, ###*p*<0.001 compared with the threshold of the vehicle-treated group at each time point. All data are presented as mean ± SEM of the paw withdrawal threshold of five to eight animals.

**Figure 4:**
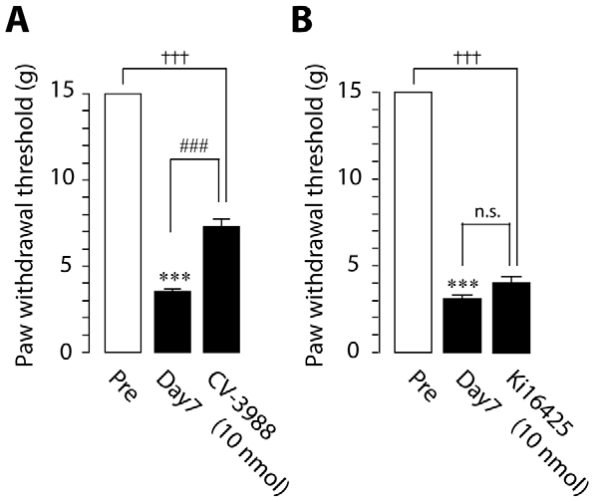
Inhibition of PAFR induces a relieving effect on existing tactile allodynia. A. The decrease in paw withdrawal threshold was attenuated by a single administration of CV-3988 (10 nmol/10 µl) (A), but not Ki16425 (10 nmol/10 µl) (B), through a catheter whose tip was positioned near the L5 DRG on day 7 after nerve injury. ***p<0.001, †††*p*<0.001 compared with pre-injury baseline (Pre). ###*p*<0.001 compared with the threshold on day 7. n.s. means “not significant”. All data are presented as mean ± SEM of the paw withdrawal threshold of five to eight animals.

To determine the role of lysophospholipid-derived lipid mediators such as PAF and LPA in nerve injury-induced tactile allodynia, we administered PAFR and LPA receptor (LPAR) antagonists to nerve-injured rats because inhibitors of PAF and LPA biosynthetic enzymes are not available. We injected the PAFR antagonist CV-3988 [29] and the LPA_1_R and LPA_3_R antagonist Ki16425 [30] near the L5 DRG and found that CV-3988 and Ki16425 significantly reduced the development of tactile allodynia (CV-3988, *p*<0.001; Ki16425, day 3: *p*<0.01, day 7: *p*<0.001, day 14: *p*<0.05) (Fig. 3A, B). By contrast, such suppressing effect of CV-3988 on allodynia development was not observed when CV-3988 was administered to the lumbar enlargement of the spinal cord (where injured DRG neurons project) (Fig. S1), suggesting that the anti-allodynic effect of the PAFR antagonist may predominantly involve PAFRs expressed in the DRG rather than in the spinal cord. In addition, CV-3988 treatment did not suppress thermal hyperalgesia (Fig. S2). Since our previous study showed that acute inhibition of cPLA_2_ produces an alleviation of existing tactile allodynia [5], we then examined whether pharmacological blockade of PAFRs and LPARs could also be effective in treating existing allodynia. A single administration of CV-3988 near the DRG 7 days after nerve injury also partially reduced the expression of tactile allodynia within 60 min (*p*<0.001) (Fig. 4A), whereas the decreased paw withdrawal threshold at day 7 after nerve injury was not reversed by a single administration of Ki16425 (Fig. 4B), consistent with a previous study [12], [31]. Alterations in motor behavior after CV-3988 and Ki16425 treatment were not observed (data not shown). These results thus indicate that the PAFR has an important role in the development and maintenance of tactile allodynia whereas the LPAR in the DRG is important only for the development of tactile allodynia.

### Lyso-PAF-acetyltransferase LPCAT2 expression in the DRG

**Figure 5:**
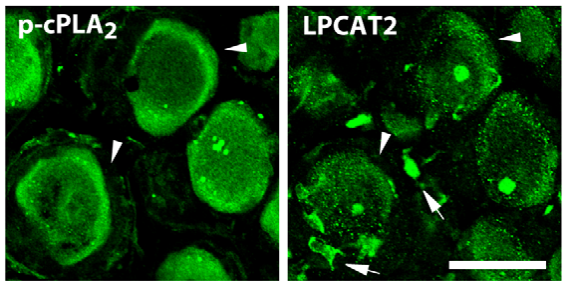
LPCAT2 is expressed in DRG neurons positive to p-cPAL_2_ following peripheral nerve injury. Immunohistochemical analysis using two adjacent DRG sections (singly immunostained one section with p-cPLA_2_ or LPCAT2 antibody) revealed that there were DRG neurons positive for both p-cPLA_2_ and LPCAT2 in the injured DRG (arrowheads) 7 days after nerve injury. Arrows indicate unknown cells that were strongly positive to LPCAT2-IR and negative to p-cPLA_2_.-IR. Scale bar, 50 µm.

Because a mechanism underlying LPAR-mediated tactile allodynia has been previously demonstrated [12], [31], in the present study we investigated the role of the PAF-PAFR system in the DRG. First, we examined whether DRG neurons with activated cPLA_2_ express the lyso-PAF-acetyltransferase LPCAT2, a critical enzyme that produces PAF [32], [33], [34]. However, it is difficult to perform double-immunolabeling of DRG sections with p-cPLA_2_ and LPCAT2 antibodies, because they were raised in the same host species (rabbit). Thus, in this experiment, we used two adjacent DRG sections and singly immunostained one section with each antibody. We observed DRG neurons that were positive for both p-cPLA_2_ and LPCAT2 in the injured DRG 7 days after nerve injury (indicated by arrowheads, Fig. 5). These results suggest that LPCAT2 and activated cPLA_2_ are co-expressed in the injured DRG. In addition, we observed many small cells that are strongly positive to LPCAT2-IR around DRG neurons, and, surprisingly, these are not overlapped with p-cPLA_2_-IR (indicated by arrows, Fig. 5). It remains unclear whether the role of LPCAT2 in these small cells in neuropathic pain, and further investigations will be needed to clarify its role.

### Upregulation of the PAFR in the DRG after peripheral nerve injury

**Figure 6:**
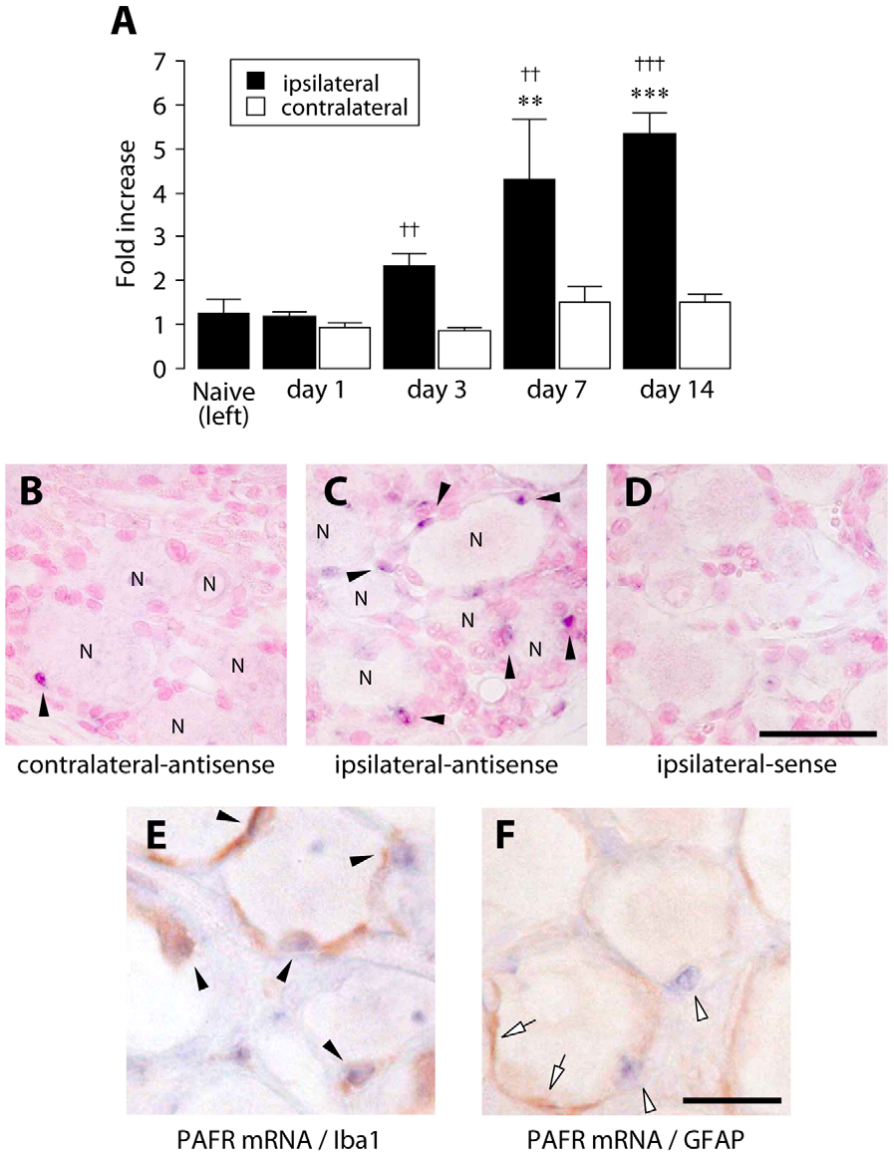
PAFR mRNA is upregulated in the DRG following peripheral nerve injury. A. Real-time PCR analysis revealed that PAFR mRNA expression in total RNA extract from the L5 DRG was markedly increased after peripheral nerve injury. The bar graphs show the average fold increase in the level of PAFR mRNA expression in the DRG compared with the mean expression level of PAFR mRNA in naïve rats. Each measurement was normalized to GAPDH content. **p<0.01, ***p<0.001 compared with naïve rats. ††*p*<0.01, †††*p*<0.001 compared with the contralateral side. All data are presented as mean ± SEM of five individual animals. B-D. DIG-labeled antisense (B: contralateral, C: ipsilateral) and sense (D: ipsilateral) probes specific for PAFR mRNA were visualized by *in situ* hybridization in the rat DRG 7 days after nerve injury. Strong PAFR mRNA signals were observed in the cells surrounding DRG neurons in the ipsilateral DRG 7 days after nerve injury. Arrowheads show PAFR mRNA-positive cells. ‘N’ indicates neuronal cells. Similar results were observed in each of three experiments. Scale bar, 50 µm. E,F. *In situ* hybridization combined with immunohistochemistry for the macrophages/microglia marker Iba1 and the satellite glia marker GFAP was performed. PAFR mRNA signals overlapped with Iba1-IR (arrowheads, E) but not with GFAP-IR (GFAP: white arrowheads, PAFR: white arrows, F). Scale bar, 25 µm.

We next examined the level of PAFR mRNA in total RNA extracts from the L5 DRG ipsilateral and contralateral to an injury to the L5 spinal nerve. We found that the expression of PAFR mRNA in the ipsilateral DRG was markedly increased after nerve injury (Fig. 6A). A significant increase was observed from day 3 after injury (*p*<0.01 compared with the value of the contralateral hindpaw) and PAFR mRNA levels peaked on day 14 (day 7: *p*<0.01, day 14: *p*<0.001 compared with the value of contralateral hindpaw and day 7: *p*<0.01, day 14: *p*<0.001 compared with that of naïve group) (Fig. 6A). The upregulation of PAFR mRNA expression on day 14 was not suppressed by CV-3988 administered for 14 days (116% of the ipsilateral DRG of vehicle-treated rats). In *in situ* hybridization analysis, we found that the intensity of PAFR mRNA signals and the number of cells with strong signal of PAFR mRNA were increased in the ipsilateral DRG 7 days after nerve injury (Fig. 6C) compared with the contralateral DRG (Fig. 6B). The expression of PAFR mRNA was increased in the cells (arrowheads) surrounding DRG neurons (indicated as ‘N’) in the ipsilateral DRG (Fig. 6C). Such specific signals of PAFR mRNA were not seen in sections hybridized with a corresponding sense probe (Fig. 6D). In addition, we performed *in situ* hybridization using another sets of probes (NM_053321 positioned at 2518-3111 bases), and similar data of PAFR mRNA were observed (data not shown). We validated the hybridization efficiency of antisense and sense probes used in this study with the spleen sections where the PAFR expression is known to be high [35] (Fig. S3). To identify the type of cells expressing PAFR, we performed *in situ* hybridization combined with immunohistochemistry for the macrophages/microglia marker Iba1 (ionized calcium-binding adapter molecule-1) and the satellite cells marker GFAP (glial fibrillary acidic protein). We showed that the PAFR mRNA signals in the injured DRG were restricted to cells labeled with Iba1 (Fig. 6E, arrowheads), but not GFAP (Fig. 6F; GFAP, arrows; PAFR mRNA, arrowheads). These results indicate that PAFR expression in the DRG is upregulated in macrophages after nerve injury.

### Attenuation of nerve injury-induced tactile allodynia in *pafr* ^−/−^ mice

**Figure 7:**
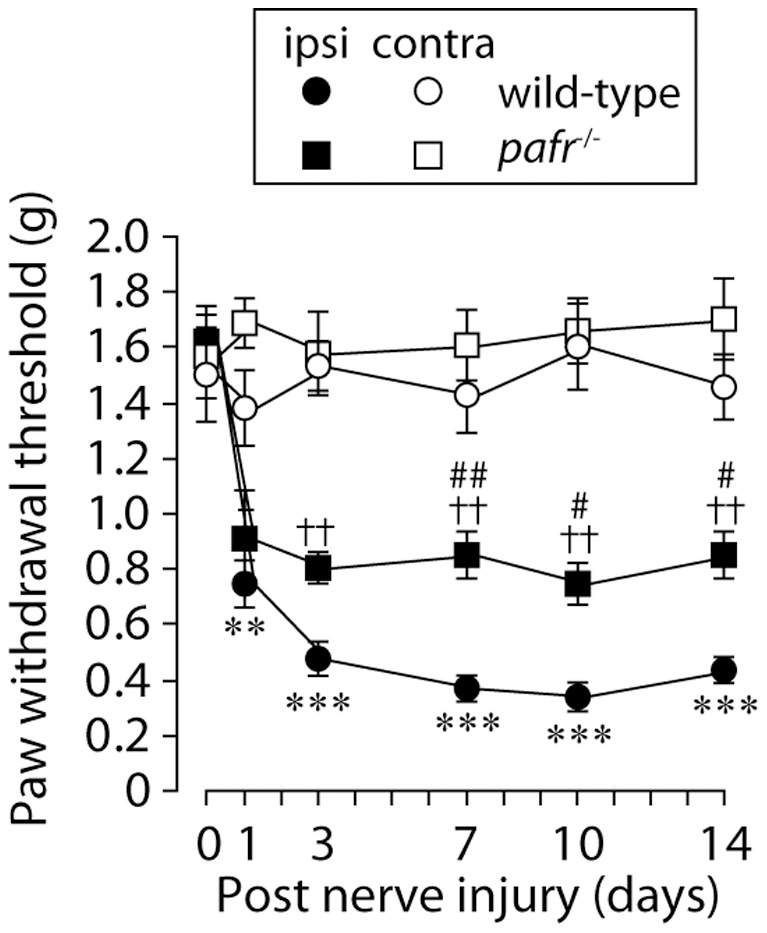
Deletion of *pafr* reduces tactile allodynia. While wild-type mice with an L5 nerve injury showed a progressively decreased paw withdrawal threshold, *pafr* ^−/−^ mice showed an attenuation of the decrease in paw withdrawal threshold after nerve injury. **p<0.01, ***p<0.001 compared with the threshold of wild-type mice on day 0. ††*p*<0.01 compared with the threshold of *pafr* ^−/−^ mice on day 0. #*p*<0.05, ##*p*<0.01 compared with the threshold of the wild-type mice at each time point. All data are presented as mean ± SEM of six to eight animals.

To precisely determine the functional relevance of PAFR, we used PAFR-deficient mice (*pafr* ^−/−^ mice) [36]. *pafr* ^−/−^ mice showed no major defects in basal mechanical sensitivity or motor coordination in the rotarod test [37]. Wild-type mice with an L5 nerve injury showed a progressively decreased paw withdrawal threshold (day 1: *p*<0.01, day 3, 7, 10 and 14: *p*<0.001) (Fig. 7). There were significant differences in the threshold between wild-type and *pafr* ^−/−^ mice (day 7: *p*<0.01, day 10 and 14: *p*<0.05) even though the threshold in *pafr* ^−/−^ mice was decreased after injury (Fig. 7). Ablation of PAFRs did not change the paw withdrawal threshold at the side contralateral to the nerve injury (Fig. 7). Thus, PAFR might be required for tactile allodynia following peripheral nerve injury.

### Reduction of upregulation of TNFα and IL-1β expression in *pafr* ^−/−^ mice

**Figure 8:**
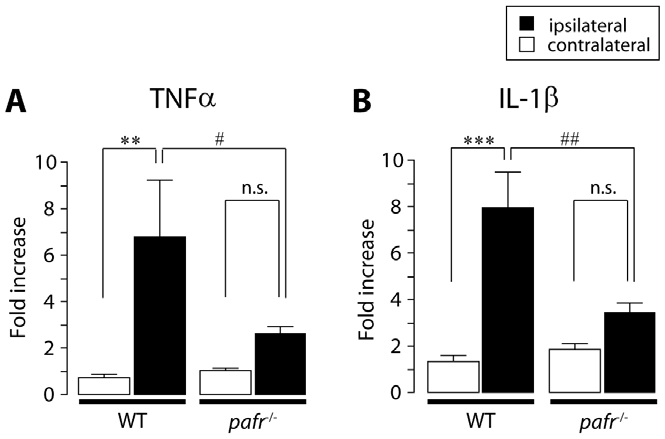
Upregulation of TNFα and IL-1β gene expression in the DRG is reduced in *pafr* ^−/−^ mice. A, B. Real-time PCR analysis demonstrated that following nerve injury, the expression of TNFα (A) and IL-1β (B) mRNAs in the ipsilateral DRG of wild-type mice was much higher than in the contralateral side on day 7. However, *pafr* ^−/−^ mice failed to show upregulation of these cytokines and there were significant differences in these expressions of the ipsilateral side between wild-type and *pafr* ^−/−^ mice. Each measurement was normalized to 18S mRNA content. **p<0.01, ***p<0.001 compared with the contralateral side of wild-type mice. #*p*<0.05, ##*p*<0.01 compared with the ipsilateral side. n.s. means “not significant”. All data are presented as mean ± SEM of six to eight animals.

TNFα and IL-1β are proinflammatory cytokines that have been reported to be upregulated in the DRG following peripheral nerve injury [16], [17], [38] and be produced via PAFRs in non-neuronal cells [39], [40]. We predicted that the loss of PAFRs could affect the levels of TNFα and IL-1β in the L5 DRG. To address this, we examined the levels of mRNA of these cytokines in the DRG after nerve injury using real-time RT-PCR. The expression levels of these mRNAs at the ipsilateral DRG of wild-type mice was much higher than at the contralateral side on day 7 (TNFα: *p*<0.01, IL-1β: *p*<0.001) (Fig. 8A, B), but *pafr* ^−/−^ mice failed to show upregulation of these cytokines and there were significant differences in these expression level of ipsilateral side between wild-type and *pafr* ^−/−^ mice (TNFα: *p*<0.05, IL-1β: *p*<0.01). Ablating PAFRs did not change cytokine expression at the contralateral DRG. These results indicate that PAFRs might contribute to tactile allodynia via TNFα and IL-1β production in the DRG following nerve injury.

### Effect of PAF injection on paw withdrawal threshold and cytokine production in the DRG

**Figure 9:**
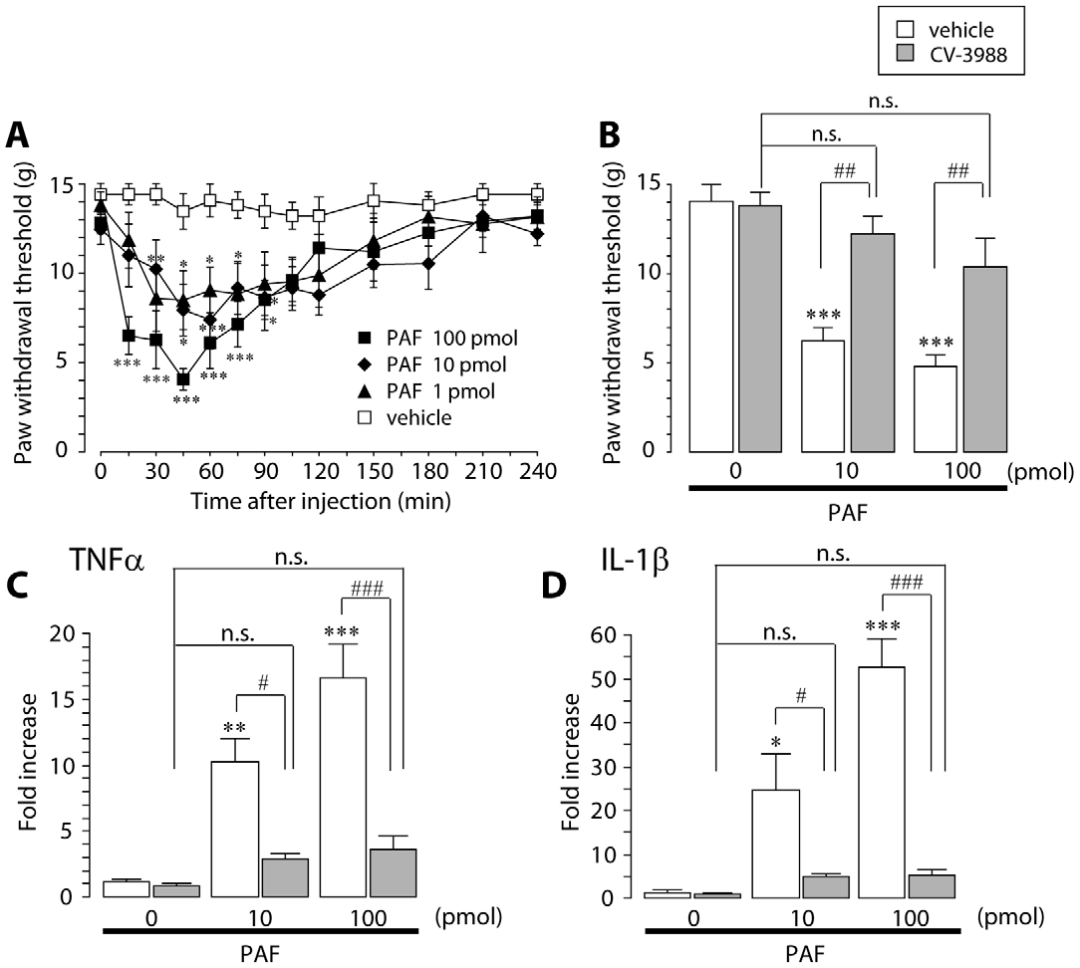
Injection of PAF near the DRG induces a decrease in the paw withdrawal threshold and upregulation of TNFα and IL-1β mRNAs. A. Paw withdrawal threshold was decreased by single administration of several different doses of PAF near the DRG in normal rats. *p<0.05, **p<0.01, ***p<0.001 compared with the threshold of the vehicle-treated group. B. The PAF-induced decrease in paw withdrawal threshold was suppressed by pretreatment with CV-3988. ***p<0.001 compared with the threshold of the vehicle-treated group. ##*p*<0.01 compared with the threshold of the PAF-treated group. n.s. means “not significant”. C, D. Real-time PCR analysis showed that administration of PAF near the DRG increased expression of TNFα (C) and IL-1β (D) mRNAs in the DRG 45 min after the administration. The bar graphs show the average fold increase in the level of TNFα and IL-1β mRNAs in the DRG compared with the mean expression level of these mRNAs in the vehicle-treated group. Each measurement was normalized to 18S mRNA content. *p<0.05, **p<0.01, ***p<0.001 compared with the vehicle-treated group. #*p*<0.05, ###*p*<0.001 compared with the PAF-treated group. n.s. means “not significant”. All data are presented as mean ± SEM of four to six animals.

We determined whether injection of PAF in the absence of nerve injury is sufficient to produce tactile allodynia and to upregulate cytokine expression in the DRG. A single injection of PAF near the DRG of naïve rats caused a decrease in the paw withdrawal threshold to mechanical stimulation in a dose-dependent manner (Fig. 9A). The threshold began to decrease 15 min after PAF injection and peaked at 45–60 min, then gradually recovered over 4 h (Fig. 9A). The decrease in the withdrawal threshold induced by PAF was inhibited by pretreatment with CV-3988 (*p*<0.01) (Fig. 9B). We then performed real-time RT-PCR analysis using the DRG of rats that had been injected with PAF. The expression of mRNAs for TNFα and IL-1β was increased 45 min after PAF injection (Fig. 9C, D), when the threshold was markedly reduced. This effect was also prevented by pretreatment with CV-3988 (Fig. 9C, D). These results suggest that administering PAF near the DRG causes a decrease in the withdrawal threshold and production of TNFα and IL-1β via PAFR.

## Discussion

In the present study, we provide the first evidence that in the DRG, PAFR is important in the pathogenesis of tactile allodynia, a major behavioral consequence of nerve injury, and are responsible for production of proinflammatory cytokines such as TNFα and IL-1β.

We have previously shown that cPLA_2_ is translocated to the plasma membrane mainly in medium- to large-diameter DRG neurons [5], but COX-1 was present around the perinuclear region of small-sized DRG neurons. In addition, our behavioral studies demonstrated that administering the COX-1 inhibitor SC-560 near the injured DRG showed no anti-allodynic effect at doses that are considered to inhibit this enzyme [25], [26]. A previous study reported that administration of SC-560 to the lumbar enlargement of the spinal cord at a higher dose than our study prevented a decrease in the paw withdrawal threshold after nerve injury [41], which seems to be partly inconsistent with our present results. However, peripheral nerve injury causes an increase in COX-1 and COX-2 expression in the spinal cord [42], [43], but not in the DRG, and a high dose of SC-560 also slightly inhibits COX-2 [21]. Thus, it is possible that the discrepancy in the effect of SC-560 on tactile allodynia might be explained by the difference in dose and region of drug administration. These results thus suggest that COX in the DRG is not primarily involved in the cPLA_2_-dependent tactile allodynia. These data may also provide a putative explanation for the resistance or controversial effects of COX inhibitors on allodynic behavior reported in both animals and patients with neuropathic pain [44].

Similar to SC-560, neither the 5-LOX inhibitor AA-861 nor the 12- and 15-LOX inhibitor baicalein showed the inhibitory effect on nerve injury-induced tactile allodynia. Products of lipoxygenases such as leukotrienes, hydroxyeicosatetraenoic acids (HETEs) and hydroperoxyeicosatetraenoic acids (HPETEs) have been implicated in mediating inflammatory nociception because they are produced during inflammation [45] and cause hyperalgesia when injected intradermally [46], [47], but so far there have been no reports that clearly show the involvement of lipoxygenases in tactile allodynia. These findings and the data presented here suggest that in the DRG, LOX does not also play a major role in nerve injury-induced tactile allodynia as in the case of COX, while we can not completely exclude the possibility that COX and LOX in the spinal cord are involved in tactile allodynia. Furthermore, because COX-1 expression was observed mainly in small-diameter DRG neurons and LOX products activate capsaicin receptors in primary sensory neurons [9], [10], COX and LOX in the DRG may participate in thermal hyperalgesia after nerve injury.

The crucial role of PAFR in neuropathic pain was demonstrated for the first time by the data obtained from the behavioral analyses using pharmacological and genetic tools. Administration of the PAFR antagonist CV-3988 near the injured DRG reduced the development and expression of nerve injury-induced tactile allodynia. By contrast, the reducing effect of the development of tactile allodynia by administration of CV-3988 to the lumbar enlargement of the spinal cord was much less than that by injection of the antagonist near the DRG. Furthermore, DRG neurons with activated cPLA_2_ also expressed the lyso-PAF-acetyltransferase LPCAT2, a critical enzyme that produces PAF [32], [33], [34]. Our data from real-time RT-PCR analyses showed that PAFR gene expression was increased in the ipsilateral DRG after nerve injury. These findings thus suggest that the PAF/PAFR system may be activated in the injured DRG and contribute to nerve injury-induced tactile allodynia, although a possible involvement of PAFR in the spinal cord can not be completely excluded [13]. Moreover, our *in situ* hybridization experiments revealed that following peripheral nerve injury a marked increase in PAFR mRNA expression was seen mainly in non-neuronal cells positive to Iba1. It is conceivable that peripheral nerve injury causes the transcriptional upregulation of PAFR in macrophages in the DRG. Alternatively, infiltration of macrophages expressing PAFR in the DRG after nerve injury might result in the upregulation of PAFR mRNA, because PAFR is expressed in macrophages [35] and is involved in macrophage chemotaxis [48] and the number of macrophages in the DRG is increased following peripheral nerve injury [49]. Interestingly, PAFR-deficient mice showed a marked suppression of the upregulation of TNFα and IL-1β expression in the injured DRG. This is consistent with the notion that macrophages are one of the major sources of these proinflammatory cytokines and regulate pain signaling [49], [50], [51], [52], although it remains unknown whether PAFR activation leads to upregulation of proinflammatory cytokine expression in macrophages.

It has been reported that TNFα [38], [53], [54], [55], [56] and IL-1β [16], [17] are upregulated in the DRG following peripheral nerve injury and are implicated in tactile allodynia. Increased synthesis and release of TNFα and IL-1β can directly modulate neuronal activity and elicit spontaneous action potential discharges in the DRG [49]. TNFα enhances tetrodotoxin-resistant Na^+^ currents in DRG neurons through TNF receptor 1 [57]. Both injured and adjacent uninjured primary afferent neurons become more sensitive to TNFα after spinal nerve ligation [18]. Similarly, IL-1β application has been shown to increase the excitability of sensory neurons by potentiating voltage-dependent Na^+^ currents [58]. Therefore, these cytokines in the injured DRG might be involved in sensitization of primary sensory neurons that link to nerve injury-induced tactile allodynia. It remains unclear whether the expression of these cytokines results from PAFR activation in non-neuronal cells in the DRG. In the future, by generating using cell type-specific PAFR-knockout mice, and by studying their phenotypes, this issue will be determined.

LPA could also be a candidate signaling molecule that is responsible for nerve injury-induced tactile allodynia. Our behavioral study demonstrated that injection of the LPAR antagonist Ki16425 near the injured DRG reduced the development, but not maintenance, of tactile allodynia, which is consistent with a previous study [12]. Because among LPARs, LPA_1_R is mainly expressed in the DRG and LPA_1_R-deficient mice attenuate nerve injury-induced tactile allodynia [12]. LPA_1_Rs mediate demyelination and upregulate the expression of the α_2_δ_1_ subunit of the voltage-gated calcium channel in the DRG [12], and these might be the mechanisms underlying LPA-dependent tactile allodynia. Furthermore, LPA increases the intracellular calcium concentration in adult DRG neurons [59] and intraplantar injection of LPA causes a nociceptive flexor response [60], suggesting that LPA could produce direct effects on primary afferent neurons and modulate nociceptive responses.

**Figure 10:**
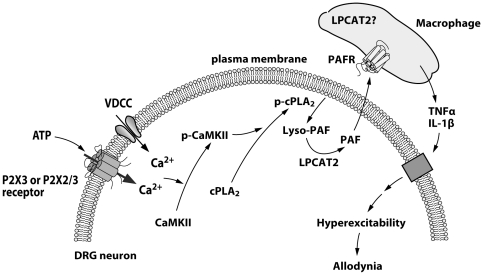
Schematic representation of the proposed mechanism underlying the PAF/PAFR system-mediated neuropathic pain after peripheral nerve injury. After peripheral nerve injury, cPLA_2_ is activated by Ca^2+^ signaling evoked by P2X3 or P2X2/3 receptors (subtype of ionotropic purinergic receptors) and voltage-gated Ca^2+^ channels (VDCC). The Ca^2+^-dependent cPLA_2_ activation involves Ca^2+^/calmodulin-dependent protein kinase II (CaMKII). cPLA_2_ supply lyso-PAF which in turn converts into PAF by the lyso-PAF-acetyltransferase LPCAT2. PAF activates PAFR expressed in macrophages. Activation of PAFR may lead to production and release of proinflammatory cytokines, TNFα and IL-1β. These cytokines may increase the excitability of DRG neurons that link to nerve injury-induced tactile allodynia.

Based on the results obtained from our present and previous studies, we proposed the following mechanism (Fig. 10). After peripheral nerve injury, cPLA_2_ is activated by Ca^2+^ signaling evoked by P2X3 or P2X2/3 receptors (subtype of ionotropic purinergic receptors) [5]. These receptors are activated by extracellular ATP, which presumably released from neighboring cells such as satellite glia. Activity of voltage-gated Ca^2+^ channels (VDCC) is also involved in cPLA_2_ activation [61]. The Ca^2+^-dependent cPLA_2_ activation is mediated by Ca^2+^/calmodulin-dependent protein kinase II (CaMKII) [61]. cPLA_2_ supply lyso-PAF, a precursor of PAF, which in turn converts into PAF by LPCAT2 (although the role of LPCAT2 expressed in unknown cells in the DRG remains unclear). PAF may be released from DRG neurons and then activates PAFR expressed in macrophages. Activation of PAFR may lead to production and release of proinflammatory cytokines. These cytokines increase the excitability of DRG neurons that link to nerve injury-induced tactile allodynia.

In summary, the present study provided the first evidence that the activation of the PAFR may be a key event in the development and maintenance of tactile allodynia and production of proinflammatory cytokines such as TNFα and IL-1β in the DRG. Thus, blocking the PAFR might be a new therapeutic target for treating neuropathic pain. Importantly, basal pain sensitivity was not altered in *pafr* ^−/−^ mice, suggesting the possibility of a therapeutic benefit of blocking PAFRs in the treatment of tactile allodynia without affecting normal pain sensitivity. As the PAFR has been implicated in other neurodegenerative disorders such as ischemia [62] and multiple sclerosis [39], interfering with PAFRs could be a therapeutic strategy widely applicable to central nervous system diseases.

## Materials and Methods

All animal experiments were conducted according to relevant national and international guidelines ‘Act on Welfare and Management of Animals’ (Ministry of Environment of Japan) and ‘Regulation of Laboratory Animals’ (Kyushu University), and under the protocols approved by the Institutional Animal Care and Use committee review panels at Kyushu University and The University of Tokyo.

### Animals

Male Wistar rats (250–270 g, Japan SLC, Shizuoka, Japan) and wild-type and *pafr* ^−/−^ mice [36] were used. Animals were housed in individual and groups of 2∼3 per cage at a temperature of 22±1°C with a 12-h light-dark cycle (light on 8:30 to 20:30), and fed food and water ad libitum.

### Neuropathic pain model

We used the spinal nerve injury model [63] with some modifications [64]: in male Wistar rats a unilateral L5 spinal nerve was tightly ligated and cut just distal to the ligature. For the experiments using mice, the left L5 spinal nerve was transected [65]. The mechanical allodynia was assessed by using calibrated von Frey filaments (0.4–15.1 g for rats, 0.02–2.0 g for mice; Stoelting, Wood Dale, IL, USA) and the paw withdrawal threshold was determined as described previously [64].

### Drug treatment

Rats were implanted with catheters for drug injection near the L5 DRG according to the method described previously [64], [65]. Under isoflurane anesthesia, a sterile 32 gauge catheter (ReCathCo, Allison Park, PA, USA) was inserted into the intrathecal space through the atlanto-occipital membrane and to the L4 or L5 DRG and externalized through the skin [5]. After the experiments, we confirmed that the tip of the catheter was positioned near the L5 DRG. Rats were injected with each drug through the catheter using a 25 µl Hamilton syringe with a 30-gauge needle once a day from day 0 (just before the nerve injury) to day 13. The drugs used in this study are listed below: SC-560 (20 nmol/10 µl, Calbiochem, San Diego, CA, USA), AA-861 (20 nmol/10 µl, Sigma, St Louis, MO, USA), baicalein (20 nmol/10 µl, Calbiochem), CV-3988 (10 nmol/10 µl, Biomol, Plymouth Meeting, PA, USA) and Ki16425 (10 nmol/10 µl, Sigma). The paw withdrawal threshold was tested 21–24 hr after the injection of each drug at 1, 3, 7 and 14 days post-injury. For the experiment in which the effect of a single administration of CV-3988 or Ki16425 on the established allodynia was examined on day 7 after nerve injury, behavioral test was performed immediately before and after the injection of CV-3988 (10 nmol/10 µl) or Ki16425 (10 nmol/10 µl). To examine the effect of PAF on pain behavior, the paw withdrawal threshold was assessed before and after a single injection of PAF near the DRG of naïve rats. Rats were injected with CV-3988 (10 nmol/10 µl) through the catheter 10 min before the injection of PAF.

### Immunohistochemistry

Rats were deeply anesthetized by pentobarbital (100 mg/kg, i.p.) and perfused transcardially with 4% paraformaldehyde. DRG sections were removed, postfixed with the same fixative, and placed in 30% sucrose solution for 24 hr at 4°C. The DRG sections (15 µm) were incubated in a blocking solution [3% normal goat serum/0.3% Triton X-100/phosphate-buffered saline (PBS) (−)] and then with anti-phospho-cPLA_2_ (anti-p-cPLA_2_) antibody (1:1000, Cell Signaling, Beverly, MA, USA), anti-COX-1 antibody (1:50, Cayman chemical, Ann Arbor, MI, USA), anti-COX-2 antibody (1∶50, Cayman chemical), and anti-LPCAT2 antibody (1∶500, [34]). Identification of neurons was performed with a marker of neurons, microtubule associated protein 2 (1∶1000, Chemicon, Temecula, CA, USA). Following incubation, the DRG sections were incubated with anti-rabbit immunoglobulin G (IgG)-conjugated Alexa Fluor 488 or anti-mouse IgG-conjugated Alexa Fluor 546 (1∶1000, Molecular Probes, Eugene, OR, USA). The sections were then analyzed by a confocal microscope (LSM510, Zeiss, Oberkochen, Germany). DRG neurons were characterized as small (<600 µm^2^), medium (600-1200 µm^2^), and large (>1200 µm^2^)-sized neurons according to their cross-sectional areas [66].

### Real-time reverse transcription-PCR

Rats and mice were deeply anesthetized with pentobarbital, perfused transcardially with PBS, and the L5 DRG was removed immediately. Total RNA from the L5 DRG was extracted by using Trisure (Bioline, Danwon-Gu, Korea) according to manufacturer's protocol and purified using RNeasy mini plus kit (QIAGEN, Valencia, CA, USA). The amount of total RNA was quantified by measuring OD_260_ using a Nanodrop spectrophotometer (Nanodrop, Wilmington, DE). For reverse transcription, 100 ng of total RNA was transferred to the reaction with Prime Script reverse transcriptase (Takara, Kyoto, Japan) and random 6-mer primers. Quantitative PCR was carried out with Premix Ex Taq (Takara) using a 7500 real-time PCR system (Applied Biosystems, Foster City, CA) according to protocol of the manufacturer, and the data were analyzed by 7500 System SDS Software 1.3.1 (Applied Biosystems) using the standard curve method. The TaqMan probe, forward primer and reverse primer used in this study were designed as follows: rat PAFR, probe, 5′-FAM- ATCTCACCGTGGCGGACCTGCTCT-TAMRA-3′, forward, 5′-CCCGTCCAAGAAACTGAATGAG-3′, and reverse, 5′-TCGCCCTCGTTGGAGTAATAGA-3′; mouse TNFα, probe, 5′-FAM-TACGTGCTCCTCACCCACACCGTCA-TAMRA-3′, forward, 5′-GTTCTCTTCAAGGGACAAGGCTG-3′, and reverse, 5′-TCCTGGTATGAGATAGCAAATCGG-3′; mouse IL-1β, probe, 5′-FAM-TGCAGCTGGAGAGTGTGGATCCCAA-TAMRA-3′, forward, 5′-GAAAGACGGCACACCCACC-3′, and reverse, 5′-AGACAAACCGCTTTTCCATCTTC-3′; rat TNFα, probe, 5′-FAM-CGTAGCCCACGTCGTA-TAMRA-3′, forward, 5′-GACCCTCACACTCAGATCATCTTCT-3′, and reverse, 5′- GGTACAGCCCATCTGCTGGTA-3′; rat IL-1β, probe, 5′-FAM-TCTCCACCTCAATGGACAGAACATAAGCCA-TAMRA-3′, forward, 5′-AAATGCCTCGTGCTGTCTGA-3′, and reverse, 5′-GTCGTTGCTTGTCTCTCCTTGTAC-3′. The primers and probe for GAPDH and 18S mRNA were obtained from Applied Biosystems.

### *In situ* hybridization

Digoxigenin (DIG)-labeled sense and antisense RNA probes were prepared from the sequence of rat *pafr* mRNA (GenBank accession number: NM_053321) positioned at 1178–1819 bases. Rats were anesthetized and perfused transcardially with Tissue Fixative (Genostaff, Tokyo, Japan) 7 day after nerve injury. DRG sections were removed and again fixed with the same fixative. Paraffin-embedded tissues (6 µm) were dewaxed with xylene and rehydrated. After proteinase K treatment (8 µg/ml, 30 min, 37°C) and acetylation by acetic anhydride (0.25%), hybridization was performed with sense and antisense probes at concentrations of 300 ng/ml at 60°C for 16 h. After hybridization, a series of washing was performed, followed by RNase treatment (50 mg/ml, 30 min, 37°C). The sections were blocked with 0.5% blocking reagent (Roche, Indianapolis, IN, USA) in Tris-buffered saline containing Tween 20 and incubated with anti-DIG alkaline phosphatase conjugate (1:1000, Roche) for 2 h at room temperature. Coloring reactions were performed with nitro blue tetrazolium chloride/5-bromo-4-chloro-3-indolyl phosphate solution (NBT/BCIP) (Sigma, St. Louis, MO, USA) overnight. The sections were counterstained with Kernechtrot stain solution (Mutoh, Tokyo, Japan) and then mounted with Malinol (Mutoh). Non-neuronal and neuronal cells with a higher violet intensity than the sections hybridized with sense probe were considered positively labeled for PAFR mRNA. For immunohistochemistry as a second staining after ISH, the sections were treated 3% hydrogen peroxide in PBS for 15 min, and Protein Block (Dako). Sections were incubated either with the anti-Iba1 rabbit polyclonal antibody (1:1000, Wako) or anti-GFAP rabbit polyclonal antibody (1:500, Dako) at 4°C for overnight. After washing with TBS, sections were incubated with biotin-conjugated goat anti-rabbit IgG (Dako) for 30 min at room temperature, followed by the addition of peroxidase conjugated streptavidin (Nichirei) for 5 min. Peroxidase activity was visualized by diaminobenzine.

### Statistical analysis

All data are presented as means ± SEM. The statistical significance of difference between values was determined by Student's *t* test or analysis of variance (ANOVA) with appropriate *post hoc* tests. A *p* value less than 0.05 was considered to be statistically significant.

## Supporting Information

Figure S1 The effect of the PAFR antagonist CV-3988 administered to the lumbar enlargement of the spinal cord on the development of nerve injury-induced tactile allodynia. The paw withdrawal threshold of tactile stimulation was examined using von Frey filaments. CV-3988 or vehicle was administered once daily for 7 days. ***p<0.001, #p<0.05 compared with pre-injury baseline (Pre). n.s. means “not significant”. All data are presented as mean ± SEM of the paw withdrawal threshold of three animals.
(0.06 MB TIF)

Figure S2 PAFR antagonist CV-3988 does not suppress the development of thermal hyperalgesia induced by injury to the L5 spinal nerve. CV-3988 (10 nmol/10 µl) was administered near the DRG once daily for 14 days after nerve injury. *p<0.05, **p<0.01. All data are presented as mean ± SEM of the paw withdrawal latency to thermal stimulus of four to five rats.
(0.05 MB TIF)

Figure S3 Validation of digoxigenin-labeled antisense (left) and sense (right) RNA probes prepared from the sequence of rat pafr mRNA (NM_053321 positioned at 1178-1819 bases) with the spleen sections. Scale bar, 25 µm.
(1.14 MB TIF)
