# Supplementary figures and images for: Role of PAF Receptor in Proinflammatory Cytokine Expression in the Dorsal Root Ganglion and Tactile Allodynia in a Rodent Model of Neuropathic Pain
Source: PLoS One. 2010 May 3;5(5):e10467. doi: 10.1371/journal.pone.0010467 (PMC2862737; doi:10.1371/journal.pone.0010467)

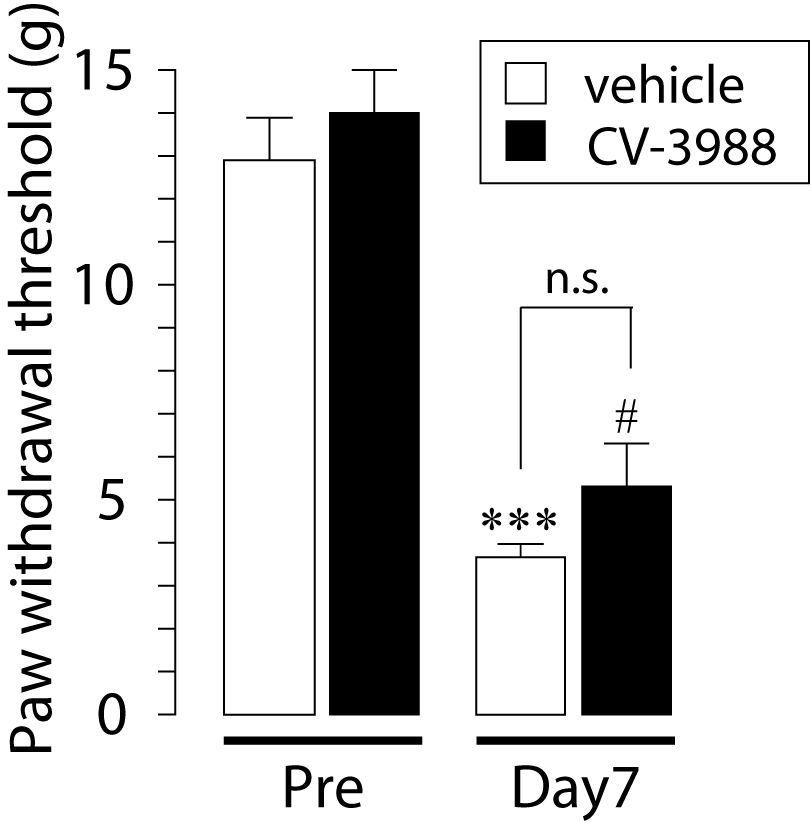

Supplement: Figure S1 — The effect of the PAFR antagonist CV-3988 administered to the lumbar enlargement of the spinal cord on the development of nerve injury-induced tactile allodynia. The paw withdrawal threshold of tactile stimulation was examined using von Frey filaments. CV-3988 or vehicle was administered once daily for 7 days. ***p<0.001, #p<0.05 compared with pre-injury baseline (Pre). n.s. means “not significant”. All data are presented as mean ± SEM of the paw withdrawal threshold of three animals. (0.06 MB TIF) [file pone.0010467.s001.tif]

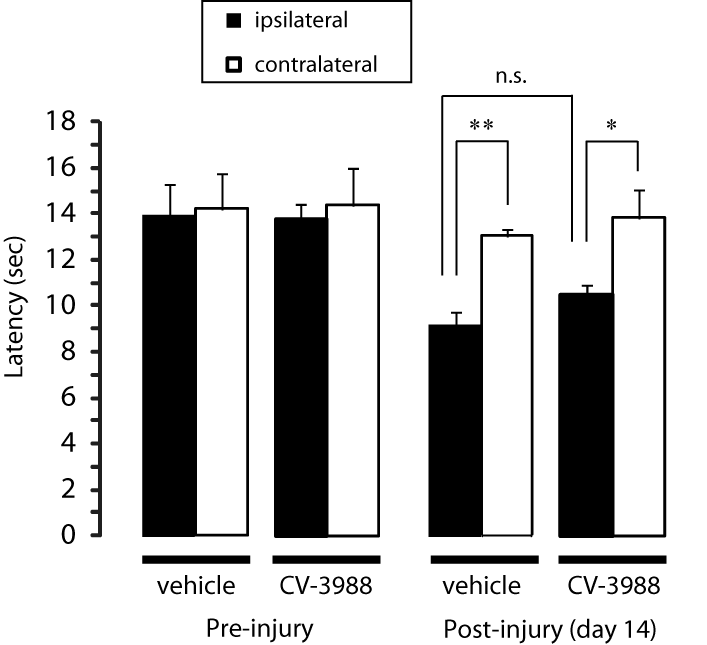

Supplement: Figure S2 — PAFR antagonist CV-3988 does not suppress the development of thermal hyperalgesia induced by injury to the L5 spinal nerve. CV-3988 (10 nmol/10 µl) was administered near the DRG once daily for 14 days after nerve injury. *p<0.05, **p<0.01. All data are presented as mean ± SEM of the paw withdrawal latency to thermal stimulus of four to five rats. (0.05 MB TIF) [file pone.0010467.s002.tif]

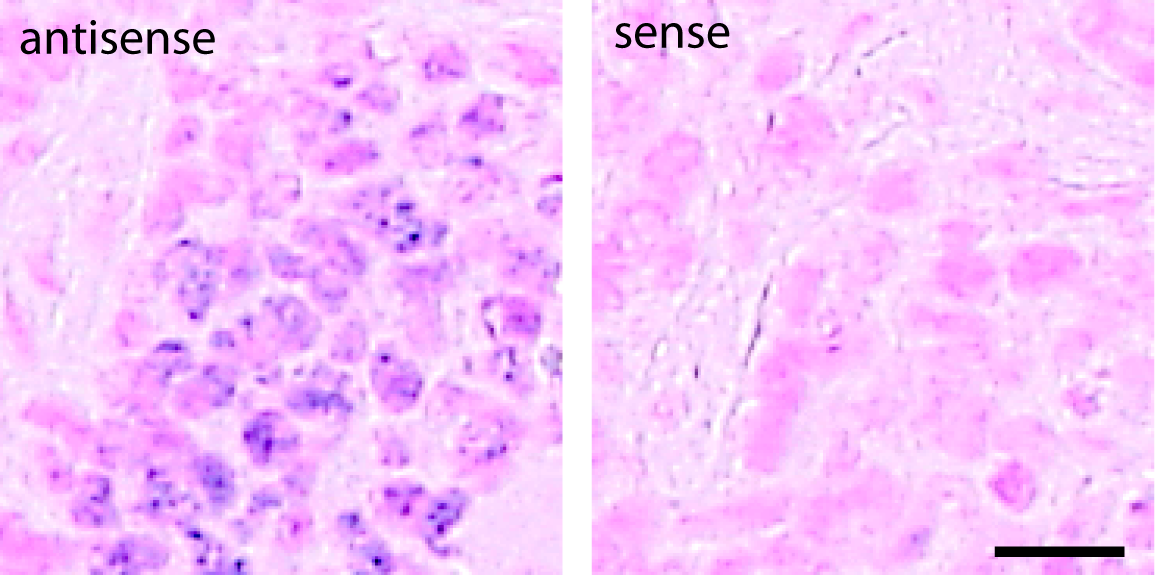

Supplement: Figure S3 — Validation of digoxigenin-labeled antisense (left) and sense (right) RNA probes prepared from the sequence of rat pafr mRNA (NM_053321 positioned at 1178-1819 bases) with the spleen sections. Scale bar, 25 µm. (1.14 MB TIF) [file pone.0010467.s003.tif]
